# Correlation between Chest X-Ray Severity in COVID-19 and Age in Mexican-Mestizo Patients: An Observational Cross-Sectional Study

**DOI:** 10.1155/2021/5571144

**Published:** 2021-04-29

**Authors:** Arturo Albrandt-Salmeron, Ruby Espejo-Fonseca, Ernesto Roldan-Valadez

**Affiliations:** ^1^Departamento de Imagenologia, Salud Digna, Av. Universidad 1338 Del Coyoacán, 04100 Mexico City, Mexico; ^2^Department of Radiology, I.M. Sechenov First Moscow State Medical University (Sechenov University), 119992 Moscow, Russia; ^3^Directorate of Research, Hospital General de Mexico “Dr Eduardo Liceaga”, 06720 Mexico City, Mexico

## Abstract

**Introduction:**

Chest X-ray (CXR) is used for the initial triage of patients with suspected COVID-19. Studies of CXR scoring in the European population found a higher score in males than in females and significantly correlated with age. Because there have not been studies in the Mexican-mestizo community, we aimed to compare the differences in CXR scores between males and females and their correlation with age after controlling comorbidities like diabetes and hypertension.

**Materials and Methods:**

A retrospective study of 1000 CXR of Mexican-mestizo patients with SARS-CoV-2 infection, confirmed by RT-PCR. Significant differences between age, age groups, symptoms, comorbidities, and CXR scores between males and females used the Mann–Whitney *U*, Chi-square tests (*χ*^2^), and Kruskal–Wallis tests. The relationship between the total CXR score and age was measured with the Spearman rank correlation coefficient (Rs); partial correlation analysis controlled the effect of symptoms, risk factors, and comorbidities.

**Results:**

The total CXR score did not show a difference between males and females grouped by age. There was a positive, low correlation between the total CXR score and age in males, Rs = 0.260, *p* < 0.001 (*N* = 616), and in females, Rs = 0.170, *p* = 0.001 (*N* = 384). Age only explained a <9% variance of CXR severity. Rs decreased its magnitude (from Rs = 0.152 to Rs = 0.046) and lost its significance (change in *p* value from *p* < 0.001 to *p* = 0.145) after controlling the effect of hypertension.

**Conclusions:**

There is no significant difference in CXR score between males and females in the Mexican-mestizo population grouped by age. Hypertension cancels the significance of CXR severity with age pointing to its role in the pathophysiology of COVID-19. Further research using stratified groups by age and gender in other populations needs to be published.

## 1. Introduction

Although computed tomography (CT) imaging is considered the most effective method for the detection of lung abnormalities, particularly in the early stage of the coronavirus disease 2019 (COVID-19) [1, 2], chest X-ray (CXR) (standard or bedside) is a useful diagnostic tool for initial triage of patients with suspected COVID-19 in the emergency department (ED) [3–5] or even at patients' homes if access to laboratory testing is limited [6]. CXR helps to monitor (“day after day”) the rapid progression of lung abnormalities in COVID-19, particularly in critical patients admitted to intensive care units [7].

In 2015, a simple five-point grading CXR scoring system was proposed for nonradiologist clinicians to facilitate the clinical grading of CXR reports in hospitalised patients with an acute respiratory infection [8]; however, that scoring system was more focused on the severity of consolidation without grading the severity between interstitial and alveolar infiltrates. In 2020, a group of radiologists validated in the European population a semiquantitative CXR scoring system for ranking the pulmonary involvement in hospitalised patients with COVID-19 based on an 18-point severity scale according to the extent and characteristics of lung abnormalities [7]. This score was based on the current knowledge of common chest CT findings in COVID-19 pneumonia (ground-glass opacity with or without patchy consolidation) [9, 10].

A previous study found a significantly higher CXR score in males than in females only in patients from 50 to 80 years; the author also reported a significant correlation between the CXR score and age in both males and females (rho = 0.205, *p* < 0.0001 for males; rho = 0.310, *p* < 0.0001 for females). There was also a difference in the scores between the age groups of each gender [11].

For this study, we hypothesised that differences by gender in the CXR score would be present in younger age groups and a lower magnitude of correlations with age. We aimed to compare the differences in CXR scores between males and females in a Mexican-mestizo population and its correlation with age.

## 2. Materials and Methods

### 2.1. Patients

This study was a retrospective single-centre observational study, HIPAA-compliant and approved by the institutional review board, which waived the required written consent. The procedures used in this study were following the institutional research committee's ethical standards and with the 1964 Helsinki Declaration and its later amendments or comparable ethical standards. The STROBE guidelines were used in reporting this study [12]. We performed a retrospective search on the department radiology information system (RIS)/picture archiving and communication system (PACS) between April and May of 2020 of chest X-ray (CXR) reports of only Mexican-mestizo patients admitted to the hospital with SARS-CoV-2 infection, confirmed by real-time reverse transcription-polymerase chain reaction (rRT-PCR) laboratory testing. Exclusion criteria applied to patients younger than 20 years, patients with no symptoms, no rRT-PCR test positivity, duplicate medical record numbers, unconfirmed results for rRT-PCR, patients with other identifiable reasons for CXR abnormalities (bacterial pneumonia, pleural effusion, lung cancer, and pulmonary oedema), unevaluable chest radiographs, and inaccessible clinical data records. Inclusion criteria considered patients older than 19 years, COVID-19 symptoms, and rRT-PCR test positivity. Patients that fulfilled the inclusion criteria were consecutively included until we completed a sample of 1000 patients. For each patient, we retrieved demographic variables such as age and gender. We additionally recorded some symptoms (cough, hyperthermia, chest pain, dyspnea, abdominal pain, and headache), the length from symptom onset to presentation in days, and four comorbidities (hypertension, diabetes mellitus (DM), type 2, asthma, and HIV infection, defined as present within one year before admission). Two board-certified radiologists independently read the radiographic images linked to the CXR reports, both with 20+ years of experience (A.A.S. and R.E.F.); however, each one could review only a partial number of CXR from the total (R.E.F. read the first 120 CXR; A.A.S. read the remaining 880 CXR) for that reason we could not report and interreader agreement. The images were anonymised, and the readers were blinded to clinical data aside from the known diagnosis of COVID-19 in all patients. The selected patients were divided into seven groups according to age: 20–29 years (group 1), 30–39 years (group 2), 40–49 years (group 3), 50–59 years (group 4), 60–69 years (group 5), 70–79 years (group 6), and ≥80 years (group 7).

### 2.2. Composite Reference Standard for COVID-19

By the time the data of this study were collected (from April to May 2020), we had followed the guidelines published on May 18^th^ 2020, by Graziadio et al. from The Centre for Evidence-Based Medicine of the University of Oxford to fulfil a temporary Composite Reference Standard (CRS) that included radiological findings to support the identification of biomarkers discriminatory for COVID-19 [13].

The CSR took into account the feasibility of use in practice. Our patients complied with the criteria for DEFINITE COVID-19 (any positive rRT-PCR result during the disease and ANY radiological evidence of pneumonia with a characteristic symptomatic presentation (sustained cough and fever) symptom of COVID-19.

### 2.3. CXR Scoring System for COVID-19 Pneumonia

We used the CXR scoring system for COVID-19 pneumonia, published by Borghesi and Maroldi [7]. For this scoring system, the lungs were divided into six zones on frontal chest projection (posteroanterior or anteroposterior projection according to the patient position) by drawing one horizontal line at the level of the inferior wall of the aortic arc and a second one above the inferior wall of the right inferior pulmonary vein. These lines divide the lungs in the right and left upper, middle, and lower zones. A score (from 0 to 3) was assigned to each zone based on the lung abnormalities detected on CXR: 0, no lung abnormalities; 1, interstitial infiltrates; 2, interstitial and alveolar infiltrates (interstitial predominance); and 3, interstitial and alveolar infiltrates (alveolar predominance). The partial score of each zone was calculated; then, the six lung zones' scores were added to obtain an overall “CXR score,” ranging from 0 to 18.

### 2.4. Statistical Analyses

We started our analyses by performing normality tests for the selected variables. The data were presented as the number and percentage (%), median, and interquartile range (IQR) if the variables were not normally distributed. We compared the age, age groups, symptoms, comorbidities, and CXR scores between males and females using the Mann–Whitney *U* and Chi-square tests (*χ*^2^). We applied the Kruskal–Wallis test to find significant differences in the CXR score between the age groups.

We evaluate the relationship between the total CXR score and age using the Spearman rank correlation coefficient (Rs). We chose the Rs because it is a nonparametric test that can be used with variables that have a nonnormal distribution [14]. We also compared the correlations reported in Italians and compared them with our population.

We used the partial correlation analysis to control the effect of symptoms, risk factors, and comorbidities [15]. Then, we evaluated two results for each partial association: the increase or decrease in the strength of the correlation coefficient and the change in statistical significance. Each correlation coefficient was interpreted as very strong (at least 0.8), moderately strong (0.6 up to 0.8), fair (0.3 up to 0.6), and poor/low (less than 0.3). Squaring *R* values represented the coefficient of determination, the proportion of variance that each two compared variables had in common [16]. We additionally tested the statistical significance of the difference between Rs coefficients of the total CXR score and age of males versus females, and with the *R* of previous studies, by converting each pair of *R* values into standard *Z* scores by using the formula [14]

(1) $$\begin{array}{c} Z_{\text{obs}}=\frac{Z_{1}-Z_{2}}{\sqrt{\left(1/\left(N_{1}-3\right)N_{1}-3\right)+\left(1/\left(N_{2}-3\right)N_{2}-3\right)}}. \end{array}$$

Observed *Z* values (*Z*_obs_) ≤ −1.96 or ≥1.96 were considered statistically significantly different. We calculated the width of the 95% confidence intervals (C.I.) of the Rs by using the formula 1.96 × standard error (SE):

(2) $$\begin{array}{c} 1.96\times \sqrt{\text{p }\left(\frac{1-\text{p}}{n}\right)}. \end{array}$$

Percentages of Rs and 95% CI were used in bar graphs for visual display of the correlation differences between males and females of our study and two additional comparisons of male versus male, female versus female, of our patients with the previous research of Borghesi and Maroldi [7].

Statistical analyses were carried out using IBM® SPSS® Statistics software version 26.0.0.1; RRID:SCR_019096 (IBM Corporation; Armonk, NY) and Microsoft Excel v16.33; RRID:SCR_016137 (Microsoft Corporation, Redmon, WA, USA). Bar graphs with 95% C.I. were drawn using SigmaPlot v14.0; RRID:SCR_003210 (Systat Software Inc., San Jose, CA, USA). Statistical significance was indicated by *p* < 0.05 (two-tailed).

## 3. Results

### 3.1. Demographics of COVID-19 Patients

We include 616 (61.6%) males and 384 (38.4%) females. Median and IQR for males were 50.00 and 23 years and 53.00 and 19 years for females. No significant difference was found between the age of males and females, Mann–Whitney *U* = 11,3340.5, *p* = 0.267. Comparing percentages between gender and age groups also did not found significant differences, *χ*^2^ (6) = 11.562, *p* = 0.072.

### 3.2. Symptoms, Risk Factors, and Comorbidities

We did not find significant differences between males and females in the reported symptoms of cough, *χ*^2^ (1) = 0.274, *p* = 0.601; hyperthermia, *χ*^2^ (1) = 0.211, *p* = 0.646; chest pain, *χ*^2^ (1) = 3.660, *p* = 0.056; dyspnea, *χ*^2^ (1) = 0.170, *p* = 0.680; and headache, *χ*^2^ (1) = 0.005, *p* = 0.945. Although there were only 10 cases of abdominal pain in man (1.6%), they represented a significant difference compared with 0 (0%) cases in females, *χ*^2^ (1) = 9.753, *p* = 0.002.

A positive history of smoking was present in 366 (59.4%) males and 181 (47.1%) females which depicted significant difference, *χ*^2^ (1) = 14.396, *p* < 0.001.

From the four comorbidities that we recovered from the medical history, none of the cases had history of HIV infection. The cases with asthma in males 2 (0.3%) and in females 2 (0.5%), as well as the cases with hypertension in males 152 (24.7%) and in females 95 (24.7%), did not represent a significant difference, *χ*^2^ (1) = 0.228, *p* = 0.633 and *χ*^2^ (1) = 0.001, *p* = 0.982, respectively.

We found 183 (29.7%) cases in males and 138 (35.9%) cases in females with DM type 2; these percentages depicted a significant difference, *χ*^2^ (1) = 4.212, *p* = 0.040.

### 3.3. Total CXR Score and Regional CXR Severity Score Differences between Males and Females

The total CXR score did not show a difference between males and females grouped by age groups; the median in all groups had a value between 2.5 and 6.0; Table 1.

**Figure 1:**
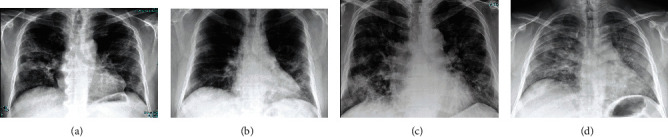
Examples of total CXR scores of patients with middle and severe COVID-19 infection. (a, b) Examples of patients with total CXR of 9 and 8, respectively. (c, d) Cases with CXR total scores in the severe range, 16 to18.

Figure 1 depicts an example of total CXR scores of patients with middle and severe COVID-19 infection.

The regional comparison of the CXR severity score at each lung's three zones revealed only a significant difference in the percentages between females and males for the right lung middle zone, Table 2.

### 3.4. Total CXR Score and Regional CXR Severity Score Differences between Age Groups

Comparing the mean ranks of the Kruskal–Wallis Test among age groups revealed significant differences for each of the six lung zones and the total CXR score (Table 3.

### 3.5. Total CXR Severity Score Correlations with Age and Partial Correlation Analyses

**Figure 2:**
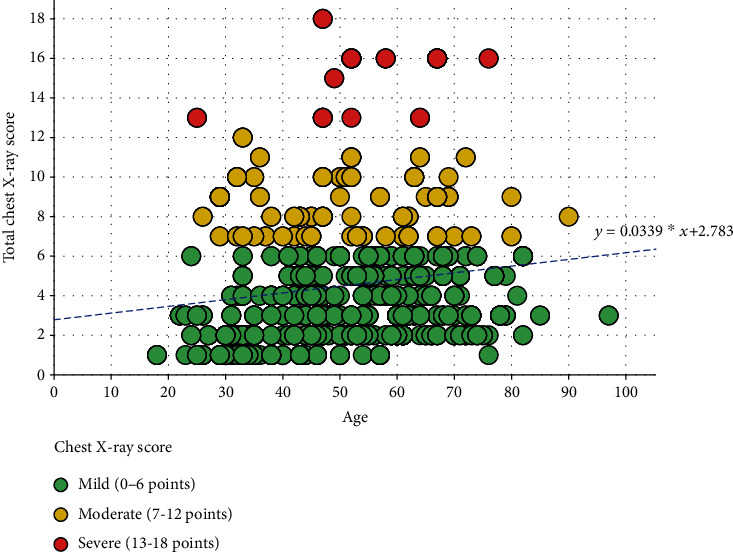
Correlation between age and the total CXR score, coloured circles, represented the grouping of cases by severity score (mild 0 to 6 points, intermediate 7 to 12 points, and severe 13 to 18 points).

There was a positive, low correlation between the total CXR score and age, Rs = 0.225, *p* < 0.001.  Figure 2 shows the correlation between age and the total CXR score.

Grouped by gender, the correlation between the total CXR score and age in males was Rs = 0.260, *p* < 0.001 (*N* = 616) and in females, Rs = 0.170, *p* = 0.001 (*N* = 384).

We performed an additional group of assessments to understand whether other variables might influence the Rs coefficient between total CXR score and age. The Rs remained in their low magnitude, without a change in its direction (positive) and significance after controlling the effect of hyperthermia, cough, chest pain, dyspnea, abdominal pain, headache; the same behaviour for the Rs was observed after controlling the effect of other variables of clinical history (smoking history, length in days from symptom onset to presentation) and comorbidity factors such as DM type 2.

We found, however, that the Rs coefficient between the total CXR score and age decreased its magnitude (from Rs = 0.152 to Rs = 0.046) and lost its significance (change in *p* value from *p* < 0.001 to *p* = 0.145) after controlling the effect of hypertension.

### 3.6. Comparison of Our Rs Correlations in Males and Females and with the Values Reported by Borghesi and Maroldi [7]

There was a trend toward significance between the correlation coefficients calculated for males and females (Rs = 0.260 and Rs = 0.170, respectively) in our study; *Z* = 1.45, *p* value = 0.074.

The comparison of the Rs observed in the male patients (Rs = 0.260) of our study versus the Rs of male patients in the study of Borghesi (Rs = 0.2050) did not show significant differences; *Z* = 0.98, *p* value = 0.164.

**Figure 3:**
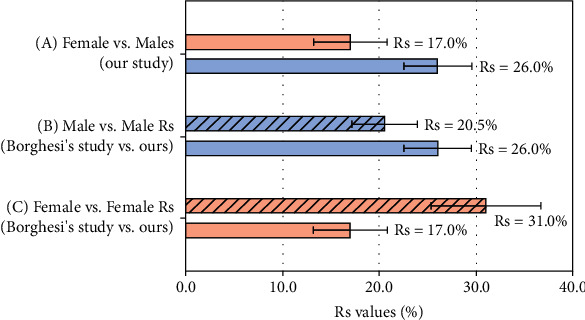
Comparison of Rs from CXR severity and age between groups. (a) Grouped bar graphs between males and females in our study (Rs = 0.260 and Rs = 0.170, respectively). (b) Comparison between males (from Borghesi's study, Rs = 0.205) vs. males (our patients, Rs = 0.260). (c) Females (from Borghesi's study, Rs = 0.310) vs. females (our patients, Rs = 0.170). The 95% confidence intervals of the bars for the Rs per cent of the females vs. females comparison did not overlap. Therefore, the percentages were significantly different, as described by their *p* value in Results.

The comparison of the Rs observed in our female patients (Rs = 0.170) versus the Rs of female patients in the study of Borghesi (Rs = 0.310) did reach significant differences; *Z* = −1.82, *p* value = 0.034.  Figure 3 show bar graphs to compare the percentages of Rs between males and females in our study and between males vs. males and females vs. females comparing our the study with the one of Borghesi et al. The 95% confidence intervals of the bars for the Rs per cent of the females vs. females comparison did not overlap with each other, and therefore, the percentages were significantly different as described by their *p* value.

## 4. Discussion

Although CXR has logistical advantages compared to CT, its clinical utility is dependent on the temporal relationship to symptom onset [17]; readers should be aware that the Fleischner Society has put forth a consensus statement on imaging recommendations for COVID-19 scenarios stratified by clinical risk of disease progression [18]. On the other hand, the American College of Radiology has also recommended that CXR in ambulatory settings be considered medically necessary [17]. To the best of our knowledge, this study is the first to examine the relationship between the severity of lung disease using the CXR severity score and the age, grouped by gender, in a Mexican-mestizo population with confirmed COVID-19. Multiple CXR grading schemes have been proposed to quantify pulmonary illness severity in COVID-19 [11, 19–21]. Of these previous studies, two were coincident in the method to assess severity; the reviews by Toussie et al. [20] and Borghesi et al. [11] proposed an independent quantification in three regions of each lung with a total score; Borghesi also included an independent analysis for males and females. The study by Reeves et al. [21] used four zones in each lung, and their study did not include separated analysis for males and females. Orsi et al. [19] used a system where each lung, as a whole, received a score of 0–4, with a maximum total score of 8 for CXR. With at least two previous studies in the literature, using the method of regions for each lung, we followed the method by Borghesi et al. [11]. However, we performed supplementary statistical analyses that allowed us to unveil additional findings.

Our study's relevance has five components: first, using a sample of 1000 patients, we did not find a significant difference in the median of age between males and females, neither in comparing age groups. These findings differ from the studies of Borghesi and Maroldi [7] and Reeves et al. [21] that studied older populations. Their patients' mean age was 65 years; our population was younger, with a median for males 50 years and 53 years for females.

Second, in the assessment of symptoms, risk factors, and comorbidities, ten cases of abdominal pain presented only in males depicted significant difference (*p* = 0.002); we also find a substantial difference in a 12.3% higher rate of smoking in males and 6.2% higher prevalence of diabetes mellitus type 2 in females. Identification of active smoking is essential, as it was found to be 11.9% more frequent in patients with a high-risk CXR score (*p* = 0.026) [21].

Third, for the Mexican-mestizo population, we did not find a significant difference in the total CXR score between males and females grouped by age groups; our findings differ from Borghesi and Maroldi [7]; they reported that in the age groups between 50 and 79 years, pulmonary involvement was significantly greater in males than in females. The significant difference in the total CXR score and for each of the six lung zones between age groups seems to be in agreement with the findings of Reeves et al. [21] that reported greater extent and severity of disease in the lower lung zones than the upper zones. However, they did not mention the statistical significance of this finding. We think the low prevalence of hypertension in our patients (below 25%) could be associated with similar patterns between males and females. Borghesi and Maroldi [7] did not analyse underlying comorbidities (such as hypertension and diabetes); these two are accepted risk factors of fatal outcomes [22, 23].

Fourth, we unveiled that three correlation coefficients between CXR with age (Rs for both genders, Rs of only male, and Rs of only females) were at the level of poor and low (Rs < 0.3), with a coefficient of determination <9%; this percentage represents that the variance explained by age is very low in its association with the severity score. Our study is the first to demonstrate that after controlling the effect of hypertension, the Rs of the CXR severity score and age decreased their magnitude and lost their significance; these findings evince the importance of hypertension in the pathophysiology of COVID-19 and its direct influence on the CXR severity score. As each additional year of age has been associated with an increase in mortality of 8% [21], this effect of hypertension deserves further research using stratified groups by age and gender.

Fifth, when we compared our findings with an Italian population, we evinced significant differences in the subgroups of the CXR severity vs. age between the patients of the study of Borghesi and Maroldi [7] and our Mexican-mestizo patients; the magnitude of the Rs in Italian females (Rs = 0.310) was 14% higher than the Rs observed in Mexican-mestizo females (Rs = 0.170). To the best of our knowledge, this fact had not been published in the literature.

Some limitations need to be acknowledged. This study was retrospective and lacked the follow-up in the CXR score. Although our patients had chest CT scans as part of their examinations, we did not report those images because a CT assessment was not within this study's scope. In April 2020, the expert panel for COVID-19 decided that there was not enough robust evidence around diagnostic accuracy of signs and symptoms and other imaging or laboratory biomarkers for these to be used as part of a CRS [13]; CT, the most efficient imaging tool in the diagnosis and follow-up of patients with COVID-19, needs that its actual diagnostic performance is verified using a reference standard [24]. Readers must be aware that the CT findings of COVID-19 may overlap with the CT findings of lung diseases caused by other pathogens [2]; typical findings of COVID-19, such as ground-glass opacities or “reversed halo” sign, are not specific for COVID-19 [25]. We decreased the patients' selection bias by selecting them in consecutive order and only those with definitive criteria for COVID-19.

We could not control the effect of other comorbidities (such as cardiovascular disease and oncologic history) or outcome (recovery versus death). Readers must be conscious that for most health systems, CXR is no longer endorsed for screening or diagnosis of COVID-19 pneumonia if the entry of patients to rRT-PCR is sufficient given that the CXR findings are nonspecific and overlap with non-COVID-19 infections [26]. Likewise, CXR is much less sensitive than laboratory checking out, given that as many as one-third of patients with COVID-19 do not exhibit abnormalities on imaging at presentation [27, 28].

Other centres can quickly reproduce our study. It used a methodology already reported by the other two studies, and we used an internationally accepted method to grade lung patterns [11, 21]. The use of research lies primarily in generalising the findings rather than in the information gained about those particular individuals. The extent to which it is wise or safe to generalise must be judged in individual circumstances. There may not be a consensus; it is reasonable to make predictions or comparisons for patients whose essential characteristics are within the original data range [29].

Several predictive clinical models of outcomes in COVID-19 have been published [30]; however, some of these articles have evinced how, although CXR severity score is associated with other products besides hospital mortality, such as predictor of intubation and continuous renal replacement therapy, this predictor power is present only in the univariate model, but it is lost in multivariate analyses [7]; future studies should assess the influence of confounders in the multivariate models that include CXR severity as a significant predictor.

In conclusion, as COVID-19 was increasingly affecting countries around the globe in the second half of 2020 and CXR is typically the first imaging test obtained in the diagnostic workup of patients with suspected COVID-19, the correlation of imaging patterns of CXR with other clinical parameters cannot be generalised still to all populations. It is still pending to unveil essential associations with the disease like hypertension and age in different communities to understand the physiopathology and treatment effects for COVID-19.

## Figures and Tables

**Table 1:** Comparison of the total CXR score between males and females grouped by age.

Age groups	Females				Males				*p* value (Mann–Whitney *U* test)
	*N*	Median	IQR		*N*	Median	IQR		
20–29 years	26	4.50	1.00	9.00	40	2.50	1.00	8.75	0.206
30–39 years	56	2.00	2.00	3.75	120	2.00	1.00	4.00	0.884
40–49 years	80	4.00	2.00	5.75	134	4.00	2.00	6.00	0.429
50–59 years	94	5.00	2.00	6.00	124	5.00	3.00	6.00	0.901
60–69 years	97	4.00	2.50	6.00	126	4.00	2.00	6.25	0.764
70–79 years	23	3.00	3.00	4.00	59	3.00	2.00	5.00	0.535
≥80 years	8	6.00	2.25	6.75	13	6.00	3.50	6.00	0.729

**Table 2:** A regional comparison of the CXR severity score between females and males at each lung's three zones revealed only a significant difference in the right lung middle zone's percentages.

Lung side	Lung zone	Gender	0 (no lung abnormalities)	1 (interstitial infiltrates)	2 (interstitial and alveolar infiltrates (interstitial predominance))	3 (interstitial and alveolar infiltrates (alveolar predominance))	*p* value (*χ*^2^)
Right	Lower	Male	2.1%	72.2%	16.1%	9.6%	0.691
		Female	1.6%	75.5%	14.6%	8.3%	
	Middle	Male	36.7%	42.7%	14.3%	6.3%	0.002
		Female	31.3%	53.6%	8.6%	6.5%	
	Upper	Male	72.6%	19.8%	5.8%	1.8%	0.316
		Female	72.9%	19.1%	6.6%	1.4%	
Left	Lower	Male	15.6%	71.3%	10.9%	2.3%	0.103
		Female	16.5%	69.2%	11.1%	3.2%	
	Middle	Male	47.6%	40.4%	9.4%	2.6%	0.541
		Female	45.8%	41.4%	8.6%	4.2%	
	Upper	Male	81.8%	13.6%	3.1%	1.5%	0.240
		Female	79.7%	15.1%	4.7%	0.5%	

**Table 3:** Comparison of the total CXR score and regional CXR severity score differences between age groups using the Kruskal–Wallis Test ranking system.

Age groups	*N*	Right lung severity score			Left lung severity score			Total CXR score
		Lower	Middle	Upper	Lower	Middle	Upper	
20–29 years	66	498.44	477.33	562.30	509.40	497.78	437.06	457.81
30–39 years	176	444.24	383.06	442.32	417.45	390.80	468.72	343.52
40–49 years	214	503.14	519.77	476.25	471.22	529.03	489.82	513.36
50–59 years	218	545.01	564.27	532.96	531.70	526.15	517.29	564.24
60–69 years	223	496.91	527.64	527.44	558.42	528.31	551.39	555.54
70–79 years	82	477.72	448.07	486.01	495.59	483.23	465.73	480.68
≥80 years	21	616.48	615.76	474.52	547.17	643.62	496.17	650.52
Kruskal–Wallis (6 df)		26.414	57.559	27.659	43.606	44.339	31.055	80.491
*p* value		<0.001	<0.001	<0.001	<0.001	<0.001	<0.001	<0.001

## Data Availability

The data used to support this study's findings are available from the corresponding author upon reasonable request.
